# Tutorial Review of N-Path Filters and Their Time-Domain Interpretation

**DOI:** 10.3390/mi17070858

**Published:** 2026-07-18

**Authors:** Xiyuan Feng, Dian Lin, Yuxiang Zhao, Jie Xiong, Wei Liu, Yunlei Zhong, Chenhao Zhuo, Yue Yin

**Affiliations:** 1 School of Integrated Circuits (School of Microelectronics), Northwestern Polytechnical University, Xi’an 710129, China; fengxy@nwpu.edu.cn (X.F.); lindian2024@163.com (D.L.); yuxiangzhao@mail.nwpu.edu.cn (Y.Z.); 13286882209@163.com (J.X.); liuwei127@nwpu.edu.cn (W.L.); 2 Key Laboratory of Multifunctional Nanomaterials and Smart Systems, Division of Advanced Materials, Suzhou Institute of Nano-Tech and Nano-Bionics, Chinese Academy of Sciences, Suzhou 215128, China; ylzhong2022@sinano.ac.cn

**Keywords:** N-path filter, linear periodically time-varying system, harmonic transfer function, time-domain interpretation, RF receiver

## Abstract

Reconfigurable radio-frequency (RF) front ends employ N-path filters to achieve digitally tunable frequency selectivity, high linearity, and low static power. However, their linear periodically time-varying (LPTV) operation complicates analysis because an input tone is translated to multiple output harmonics. This tutorial review synthesizes the principal methods for analyzing N-path filters, comparing continuous-time window function analysis, discrete-time ordinary differential equation (ODE) modeling, and adjoint network methods. We evaluate and compare their underlying assumptions, outputs, and computational burdens. Additionally, we present an educational time-domain interpretation based on orthogonal sine/cosine excitation. This viewpoint connects capacitor averaging and path-to-path phase cancellation with harmonic transfer functions (HTFs). Rather than replacing rigorous HTF formulations, this interpretation provides a physically intuitive explanation for the fundamental coefficient $H_{0}\left(f\right)$ and the gain-null condition at $f_{\mathrm{in}}=kNf_{s}$. The numerical integration of the switched-RC equations serves as a consistency check. For a four-path example with Γ=τ/(RC)=0.02, the numerical values of $|H_{0}\left(f_{s}\right)|$ and $|H_{0}\left(2f_{s}\right)|$ differ from the intuitive limits by less than 0.001 dB. The residual responses at $4f_{s}$ and $8f_{s}$ are −49.95 dB and −55.97 dB, respectively. Finally, we extend the orthogonal-excitation relationship to extract higher-order HTFs. This tutorial synthesis clarifies how these established analytical methods relate and guides selection for specific applications.

## 1. Introduction

N-path filters implement frequency-selective radio-frequency (RF) functions by periodically commutating passive resistor–capacitor (RC) branches [1,2,3,4,5,6]. Their center frequency is set primarily by the switching clock, making them attractive for software-defined and multiband receivers [7,8,9,10,11,12,13,14,15,16,17,18,19,20,21]. However, unlike linear time-invariant (LTI) networks, an N-path filter operates as a linear periodically time-varying (LPTV) system. A sinusoidal input at frequency *f* generates frequency-translated components at $f+kf_{s}$, where $f_{s}$ is the switching frequency, and *k* is an integer [22,23,24,25]. Consequently, a single transfer function cannot describe the circuit; instead, a set of harmonic transfer functions (HTFs) is required [11,12,26,27,28,29].

Several analytical frameworks have been developed to model these systems. Continuous-time window function formulations provide a complete description but require substantial algebra and frequency-domain convolutions [27]. Discrete-time ordinary differential equation (ODE) methods yield compact sampled transfer functions, although they do not describe every translated output component [26,28]. Adjoint network methods reduce the derivation to an equivalent impulse response problem, offering an efficient route to sampled responses [30]. Although these approaches are mathematically consistent, their differing assumptions and output formats complicate direct comparison for researchers entering the field.

This article is structured as a tutorial review rather than an original research paper. Our first objective is to place continuous-time, discrete-time, and adjoint network methods into a unified LPTV framework, comparing their capabilities and limitations [26,27,28,30]. Our second objective is to present an educational time-domain interpretation based on orthogonal sine/cosine excitation. This viewpoint visualizes the physical mechanisms behind the fundamental coefficient $H_{0}\left(f\right)$ and the gain nulls at $f_{\mathrm{in}}=kNf_{s}$. We treat this time-domain picture as an intuitive visualization of LPTV behavior rather than a replacement for rigorous HTF analysis. To support this interpretation, we present a normalized numerical integration of the switched-RC equations and extract both fundamental and higher-order HTFs.

The remainder of this article is structured as follows: Section 2 introduces the general LPTV model and the N-path cancellation principle. Section 3 reviews continuous-time, discrete-time, and adjoint-network methods and then provides the proposed time-domain interpretation and its numerical validation. Finally, Section 4 summarizes the main conclusions and guides the selection of each method based on application requirements.

## 2. LPTV Modeling and N-Path Principle

### 2.1. N-Path Filters as LPTV Systems

**Figure 1:**
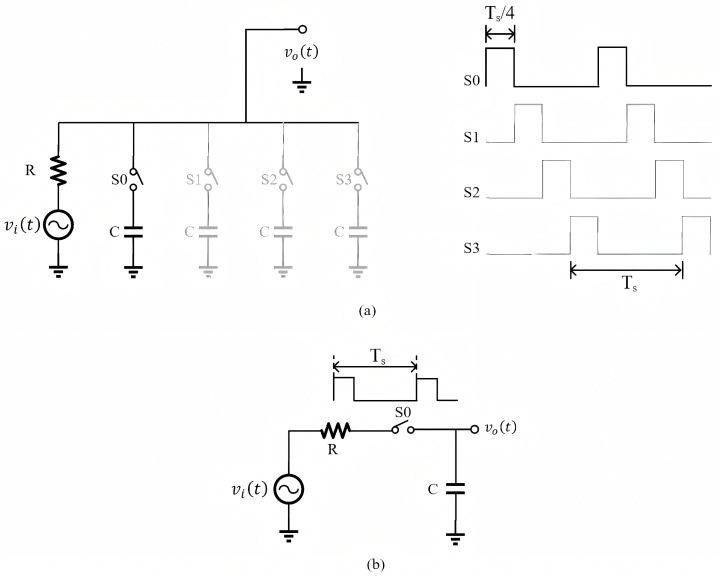
(**a**) Typical structure of SE four-path filter. (**b**) SE kernel.

Figure 1a shows a single-ended (SE) four-path filter. The circuit consists of four periodically activated switched-RC branches, one of which is shown in Figure 1b. The ideal switches are driven by non-overlapping clock phases with period $T_{s}$. For the first path, whose switch is on for $T_{s}/4$, the capacitor voltage satisfies

(1) $$\begin{array}{cc} v_{i}\left(t\right) & =v_{o}\left(t\right)+RC\frac{dv_{o}\left(t\right)}{dt},\;nT_{s}<t\leq \left(n+\frac{1}{4}\right)T_{s}\\ v_{o}\left(t\right) & =v_{o}\left[\left(n+\frac{1}{4}\right)T_{s}\right],\;\left(n+\frac{1}{4}\right)T_{s}<t\leq \left(n+1\right)T_{s}. \end{array}$$

Deriving a frequency-domain description directly from this piecewise equation is cumbersome [26,27,28]. The network is not LTI because the switch state depends on absolute time. It is instead an LPTV system with period $T_{s}$, and its impulse response satisfies

(2) $$h\left(t,\tau \right)=h\left(t+T_{s},\tau \right).$$

**Figure 2:**
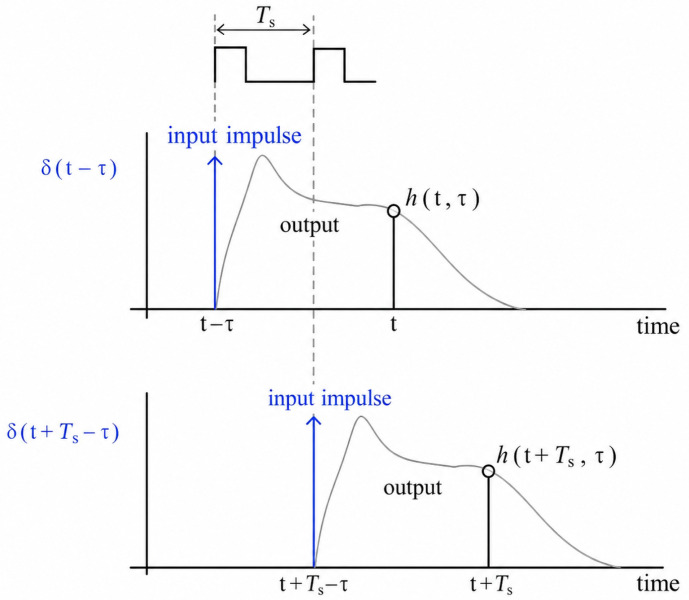
Impulse response $h\left(t,\tau \right)$ and $h\left(t+T_{s},\tau \right)$ for LPTV system.

Here, h(t,τ) denotes the response observed at time *t* to an impulse applied at t−τ. Because the clock waveforms repeat every $T_{s}$, shifting the observation time and the switching state by one period leaves the response unchanged. This periodicity is illustrated in Figure 2.

To define the frequency-domain response of an LPTV system, consider the complex exponential input $x\left(t\right)=e^{j2\pi ft}$. The corresponding output is obtained from

(3) $$\begin{array}{cc} y(t) & =\int_{0}^{\infty }h\left(t,\tau \right)e^{j2\pi f(t-\tau )}d\tau \\ \; & =e^{j2\pi ft}\int_{0}^{\infty }h\left(t,\tau \right)e^{-j2\pi f\tau }d\tau . \end{array}$$

A time-dependent transfer function can then be defined as

(4) $$H\left(f,t\right)=\frac{\mathrm{Response}\mathrm{to}\;e^{j2\pi ft}}{e^{j2\pi ft}}.$$

Using the periodicity of h(t,τ) gives

(5) $$\begin{array}{cc} H(f,t) & =\int_{0}^{\infty }h\left(t,\tau \right)e^{-j2\pi f\tau }d\tau \\ \; & =\int_{0}^{\infty }h\left(t+T_{s},\tau \right)e^{-j2\pi f\tau }d\tau \\ \; & =H(f,t+T_{s}). \end{array}$$

Because H(f,t) is periodic in *t* with period $T_{s}$, it admits the Fourier-series expansion

(6) $$H\left(f,t\right)=\sum_{k=-\infty }^{\infty }H_{k}\left(f\right)e^{j2\pi kf_{s}t},\;f_{s}=\frac{1}{T_{s}}.$$

The Fourier coefficients are the harmonic transfer functions

(7) $$H_{k}\left(f\right)=\frac{1}{T_{s}}\int_{0}^{T_{s}}H\left(f,t\right)e^{-j2\pi kf_{s}t}\;dt.$$

Accordingly, the output can be written as [29]

(8) $$H\left(f,t\right)e^{j2\pi ft}=\sum_{k=-\infty }^{\infty }\underset{\mathrm{harmonic}\;\mathrm{gain}}{\underset{︸}{H_{k}\left(f\right)}}\underset{\mathrm{translated}\;\mathrm{component}}{\underset{︸}{e^{j2\pi (f+kf_{s})t}}}.$$

An LPTV system can therefore be characterized by either h(t,τ) or the set $\left\{H_{k}\left(f\right)\right\}$. In contrast to an LTI system, for which a tone remains at the same frequency, an LPTV system translates an input at *f* to the family of frequencies $f+kf_{s}$. Each coefficient $H_{k}\left(f\right)$ specifies the complex gain from the input tone at *f* to the output component at $f+kf_{s}$.

### 2.2. N-Path Principle

For an LTI system, the transfer function can be obtained from the Fourier transform of its impulse response. Direct evaluation of the two-time impulse response of an LPTV circuit is usually impractical. Existing N-path analyses instead formulate a time-domain relation between the input and output, for example,

(9) $$X\left[v_{o}\left(t\right),\frac{d}{dt}v_{o}\left(t\right)\right]=Y\left[v_{o}\left(t\right),v_{i}\left(t\right)\right].$$

where *X* and *Y* denote algebraic or differential operators acting on $v_{o}\left(t\right)$ and $v_{i}\left(t\right)$. Fourier transformation then gives

(10) $$\begin{array}{cc} F\{X\left[v_{o}\left(t\right),\frac{d}{dt}\;v_{o}\left(t\right)\right]\} & =F\{Y\left[v_{o}\left(t\right),v_{i}\left(t\right)\right]\}\\ \Rightarrow v_{o}\left(f\right) & =Z[v_{i}\left(f\right)]. \end{array}$$

where Z[·] denotes the resulting frequency-domain operator. Equation (10) compactly represents the frequency conversion relation of the LPTV system.

The N-path principle further reduces the analysis burden. Once the HTFs of a single switched branch are known, the response of the complete N-path network follows from the relative time shifts among the clock phases [26,27,28,30]. For the input $x\left(t\right)=e^{j2\pi ft}$, Equation (8) gives

(11) $$\begin{array}{cc} y(t) & =\sum_{k=-\infty }^{\infty }H_{k}\left(f\right)e^{j2\pi (f+kf_{s})t}\\ \; & =\left[\sum_{k=-\infty }^{\infty }H_{k}\left(f\right)e^{j2\pi kf_{s}t}\right]\;e^{j2\pi ft}. \end{array}$$

If the switching waveform is delayed by $t_{o}$, the corresponding output becomes

(12) $$y_{t_{o}}\left(t\right)=\sum_{k=-\infty }^{\infty }H_{k}\left(f\right)e^{-j2\pi kf_{s}t_{o}}e^{j2\pi (f+kf_{s})t}.$$

**Figure 3:**
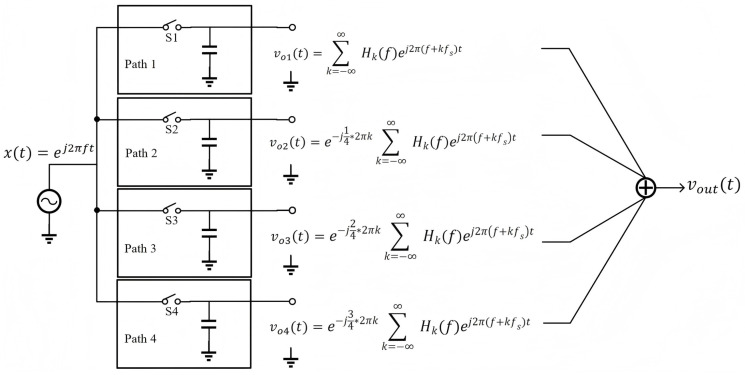
Output $v_{out}\left(t\right)$ of four-path filter is formed by four time-shifted path outputs.

**Figure 4:**
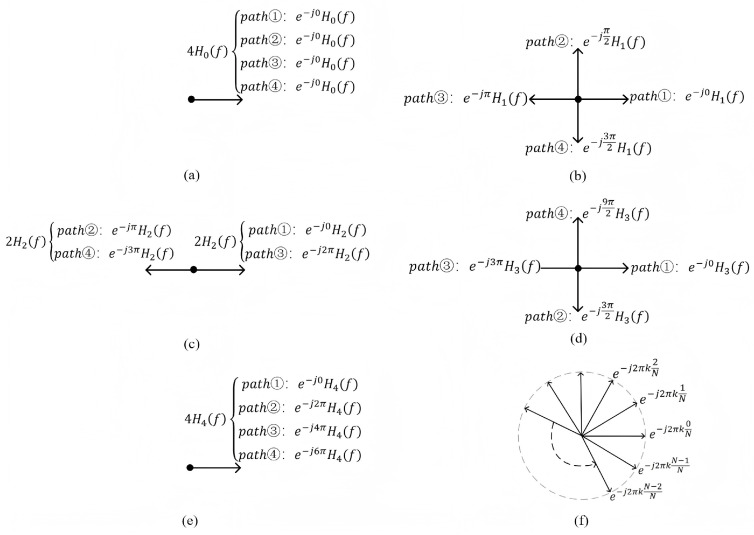
(**a**–**e**) Vector sums for zeroth- through fourth-order HTFs of four-path filter. (**f**) General *k*th-order vector sum for N-path filter.

Equation (12) shows that a delay in the switching waveform multiplies the *k*th translated component by $e^{-j2\pi kf_{s}t_{o}}$. For a four-path filter, the branch outputs in Figure 3 are separated by $T_{s}/4$. The vector sums in Figure 4 therefore cancel the coefficients whose harmonic index is not an integer multiple of four. More generally, for an N-path network,

(13) $$H_{k}^{\left(N\right)}\left(f\right)=\left\{\begin{array}{cc} NH_{k,\mathrm{SE}}\left(f\right), & k=mN,\;m\in Z,\\ 0, & \mathrm{otherwise}. \end{array}\right.$$

Equation (13) is the N-path principle. It shows that the complete response can be constructed from a single-path analysis together with the deterministic phase shifts of the clock phases.

## 3. Analysis Methods for N-Path Filters

This section reviews three established analytical methods for modeling reconfigurable N-path filters. The first method uses state-space equations and window functions to derive a continuous-time HTF description. The second method solves the switched-RC ODE at discrete switching instants to find the sampled response. The third method leverages sampling equivalence and the adjoint network to obtain an equivalent LTI impulse response. The final subsection presents our educational time-domain interpretation alongside quantitative consistency checks. The comparison focuses on the mathematical representations, underlying assumptions, and frequency conversion behaviors captured by each technique.

### 3.1. Continuous-Time Analysis Using a Window Function

**Figure 5:**
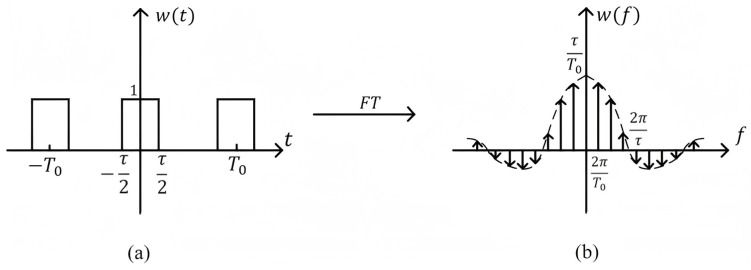
(**a**) Window function $w\left(t\right)$ and (**b**) its Fourier transform $w\left(f\right)$.

The main difficulty in transforming Equation (1) is its piecewise definition. Soer et al. introduced a periodic window function w(t), shown in Figure 5, to select the interval during which a given switch is on [27]. Multiplication by w(t) in the time domain becomes convolution with its line spectrum in the frequency domain:

(14) $$\begin{array}{cc} F(v(t)\cdot w(t)) & =\int_{-\infty }^{+\infty }w\left(f\right)\cdot v\left(f-\bar{f}\right)d\bar{f}\\ \; & =\sum_{n=-\infty }^{+\infty }w\left(f_{0}\right)\cdot v\left(f-nf_{0}\right)\\ \; & =\sum_{n=-\infty }^{+\infty }\frac{\sin(\pi nf_{0}\tau )}{n\pi }\cdot v\left(f-nf_{0}\right)\\ \; & =\sum_{n=-\infty }^{+\infty }\frac{e^{j\pi nf_{0}\tau }-e^{-j\pi nf_{0}\tau }}{2jn\pi }\cdot v\left(f-nf_{0}\right). \end{array}$$

In the state-space formulation of [27], the *k*th window has width $\tau_{k}=\sigma_{k}-\sigma_{k-1}$ and a phase term that accounts for the location of the window center. A switching-moment transfer function $G_{k}\left(f\right)$ first relates the state variables at discrete switching instants. Periodic impulse trains then extend this relation to continuous time, yielding all translated output components. Table 1 summarizes the main steps.

### 3.2. Discrete-Time Analysis Using ODEs

The method in [26], summarized in Table 2, solves the switched-RC ODE at the relevant switching instants. It avoids the explicit window function convolution and yields a compact sampled transfer function. This result is closely related to the switching-moment function $G_{0}\left(f\right)$ in [27]. Its principal limitation is that the sampled relation directly characterizes the same-frequency component, corresponding to $H_{0}\left(f\right)$, but does not by itself provide the complete set of gains from $f_{in}$ to $f_{in}+kf_{s}$.

### 3.3. Analysis Using an Adjoint Network

Both preceding approaches require solving differential equations. The adjoint network method exploits a sampling property of LPTV systems: when the output is sampled synchronously with the switching period, all components at $f+kf_{s}$ fold to the same discrete-time frequency. The sampled LPTV response can therefore be represented by an equivalent LTI transfer function, which substantially simplifies the calculation.

To see this, excite the LPTV system with $x\left(t\right)=e^{j2\pi ft}$. The output components are

(15) $$\begin{array}{cc} y_{0}\left(t\right) & =H_{0}\left(f\right)e^{j2\pi ft}\\ y_{1}\left(t\right) & =H_{1}\left(f\right)e^{j2\pi (f+f_{s})t}\\ y_{2}\left(t\right) & =H_{2}\left(f\right)e^{j2\pi (f+2f_{s})t}\\ \; & \vdots \\ y_{k}\left(t\right) & =H_{k}\left(f\right)e^{j2\pi (f+kf_{s})t} \end{array}$$

Sampling these components at $t=nT_{s}$ folds every frequency $f+kf_{s}$ to the same discrete-time frequency, so

(16) $$y\left(nT_{s}\right)=e^{j2\pi fnT_{s}}\sum_{k=-\infty }^{\infty }H_{k}\left(f\right).$$

The equivalent sampled LTI transfer function is therefore

(17) $$H_{eq}\left(f\right)=\sum_{k=-\infty }^{\infty }H_{k}\left(f\right).$$

**Figure 6:**
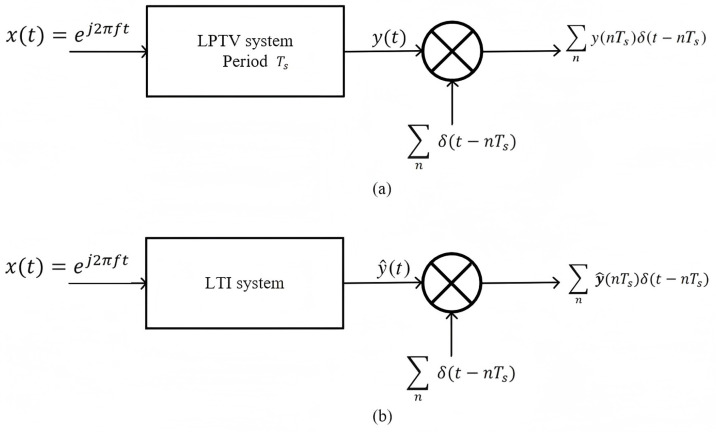
(**a**) An LPTV system (varying with period $T_{s}$) whose output is sampled at $f_{s}=1/T_{s}$. (**b**) An LTI system whose output is sampled at $f_{s}$.

The same result follows directly from the Fourier-series representation in Figure 6 [29]:

(18) $$y\left(t\right)=\sum_{k=-\infty }^{\infty }H_{k}\left(f\right)e^{j2\pi (f+kf_{s})t}.$$

The corresponding samples are

(19) $$\begin{array}{cc} y(nT_{s})\; & =\sum_{k=-\infty }^{\infty }H_{k}\left(f\right)e^{j2\pi \left(f+kf_{s}\right)nT_{s}}\\ \; & =e^{j2\pi fnT_{s}}\sum_{k=-\infty }^{\infty }H_{k}\left(f\right), \end{array}$$

The equivalent LTI system is excited by the same complex exponential $x\left(t\right)=e^{j2\pi ft}$. The output $\hat{y}\left(t\right)$ is sampled with the same period $T_{s}$ as shown in Figure 6b. Its output is

(20) $$\hat{y}\left(t\right)=e^{j2\pi ft}H_{eq}\left(f\right).$$

The corresponding samples are

(21) $$\hat{y}\left(nT_{s}\right)=e^{j2\pi nfT_{s}}H_{eq}\left(f\right)=y\left(nT_{s}\right).$$

Thus, the sampled response can be found from an equivalent impulse response $h_{eq}\left(t\right)$ and its Fourier transform. Reference [30] shows that $h_{eq}\left(t\right)$ can be efficiently obtained from the adjoint network.

**Figure 7:**
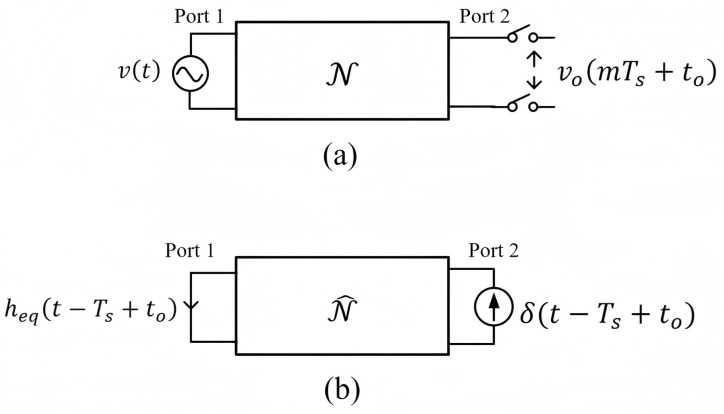
(**a**) A two-port LPTV network N and (**b**) its adjoint network $\hat{N}$.

**Figure 8:**
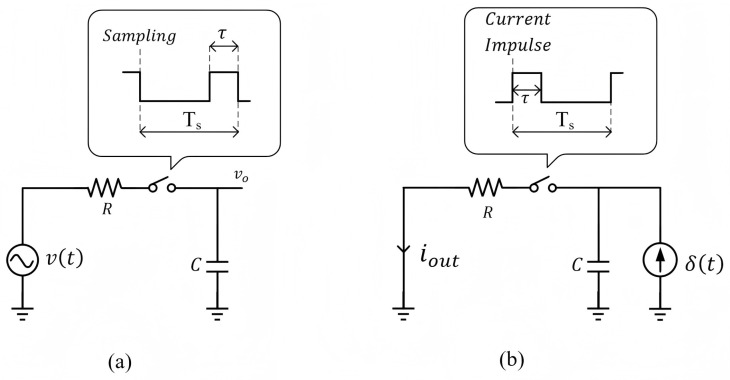
(**a**) The switched-RC network. (**b**) The adjoint network.

Consider the two-port LPTV network N in Figure 7. Port 1 is driven by v(t), and the output at port 2 is sampled at $mT_{s}+t_{o}$. In the adjoint network $\hat{N}$, a delayed impulse is applied at port 2, and the response observed at port 1 gives the equivalent impulse response of the original sampled network [30]. For the switched-RC branch in Figure 8, the impulse initializes the capacitor voltage according to

(22) $$v_{c}\left(0_{+}\right)=\frac{\int_{-\infty }^{0_{+}}i\left(t\right)dt}{C}=\frac{\int_{-\infty }^{0_{+}}\delta \left(t\right)dt}{C}=\frac{1}{C}.$$

At $t=0^{+}$, the switch is on, and the capacitor discharges through *R* with time constant RC. During the on interval 0<t<τ, the output current is

(23) $$i_{out}\left(t\right)=\frac{1}{RC}e^{-t/(RC)},\;0<t<\tau .$$

Thus, $i_{out}\left(0^{+}\right)=1/\left(RC\right)$, and $i_{out}\left(\tau^{-}\right)=\beta /\left(RC\right)$, where

(24) $$\beta =e^{-\tau /(RC)}.$$

The switch is then opened, and the current remains zero until the next activation. Because the capacitor retains its charge during the off interval, each subsequent waveform is a delayed and scaled copy of the first one. The recursive relation is

(25) $$i_{out}\left(t\right)=i_{0}\left(t\right)+\beta i_{out}\left(t-T_{s}\right).$$

Taking the Fourier transform gives

(26) $$i_{out}\left(f\right)=\frac{i_{0}\left(f\right)}{1-\beta e^{-j2\pi fT_{s}}}.$$

The first-period waveform is

(27) $$i_{0}\left(t\right)=\frac{1}{RC}\left[e^{-t/(RC)}u\left(t\right)-e^{-t/(RC)}u\left(t-\tau \right)\right],$$

which can equivalently be written with $\beta =e^{-\tau /(RC)}$ as a truncated exponential. Its Fourier transform is

(28) $$i_{0}\left(f\right)=\frac{1}{1+j2\pi fRC}\left(1-\beta e^{-j2\pi f\tau }\right).$$

Therefore,

(29) $$H_{eq}\left(f\right)=\frac{1}{1+j2\pi fRC}\frac{1-\beta e^{-j2\pi f\tau }}{1-\beta e^{-j2\pi fT_{s}}}.$$

**Figure 9:**
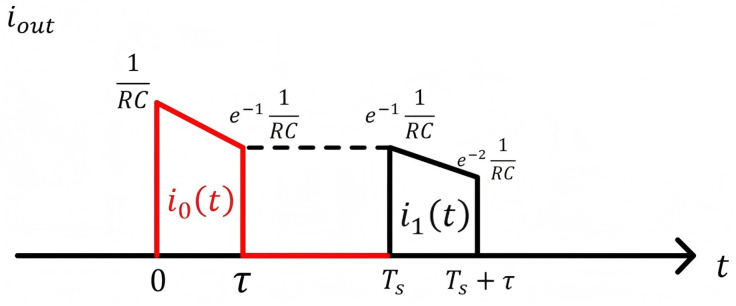
Impulse response current $i_{\mathrm{out}}\left(t\right)$.

This expression is identical to the sampled transfer function in Table 2. The resulting impulse-response current is shown in Figure 9.

### 3.4. Time-Domain Interpretation Based on Orthogonal Excitation

**Figure 10:**
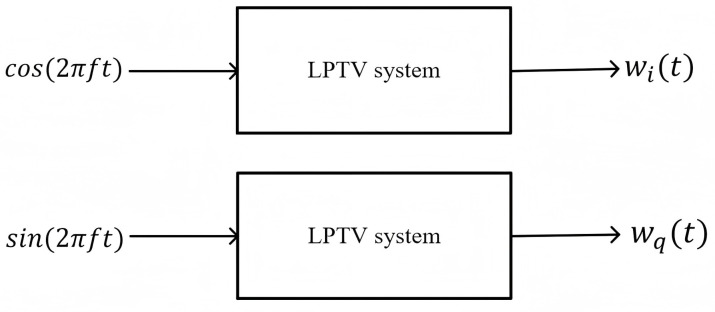
The same LPTV system with inputs of $\cos\left(2\pi ft\right)$ and $\sin\left(2\pi ft\right)$, respectively.

Although the preceding methods provide rigorous transfer functions, their algebraic complexity can obscure the physical mechanisms of capacitor averaging and path-to-path phase cancellation. To address this, we present an educational interpretation based on orthogonal excitations. Specifically, we drive the LPTV system separately with cos(2πft) and sin(2πft), as illustrated in Figure 10, and we denote the corresponding outputs as $w_{i}\left(t\right)$ and $w_{q}\left(t\right)$.

The outputs $w_{i}\left(t\right)$ and $w_{q}\left(t\right)$ can be expressed as

(30) $$\begin{array}{cc} w_{i}\left(t\right) & =\frac{1}{2}\left[H\left(f,t\right)e^{j2\pi ft}+H\left(-f,t\right)e^{-j2\pi ft}\right]\\ w_{q}\left(t\right) & =\frac{1}{2j}\left[H\left(f,t\right)e^{j2\pi ft}-H\left(-f,t\right)e^{-j2\pi ft}\right] \end{array}$$

Combining the two responses gives

(31) $$\left[w_{i}\left(t\right)+jw_{q}\left(t\right)\right]e^{-j2\pi ft}=H\left(f,t\right).$$

The real and imaginary parts of H(f,t) are therefore

(32) Re[H(f,t)]=wi(t)cos(2πft)+wq(t)sin(2πft)

and

(33) Im[H(f,t)]=wq(t)cos(2πft)−wi(t)sin(2πft).

The Fourier expansion of H(f,t) is

(34) $$H\left(f,t\right)=\sum_{k=-\infty }^{\infty }H_{k}\left(f\right)e^{j2\pi f_{s}kt},\;f_{s}=\frac{1}{T_{s}}.$$

The fundamental same-frequency coefficient is the period average

(35) $$H_{0}\left(f\right)=\frac{1}{T_{s}}\int_{t}^{t+T_{s}}H\left(f,\tau \right)d\tau .$$

This orthogonal-excitation framework can extract any harmonic coefficient $H_{k}\left(f\right)$ in addition to the fundamental response $H_{0}\left(f\right)$. Substituting $H\left(f,t\right)=\left[w_{i}\left(t\right)+jw_{q}\left(t\right)\right]e^{-j2\pi ft}$ into the Fourier-series definition yields

(36) $$H_{k}\left(f\right)=\frac{1}{T_{s}}\int_{t_{0}}^{t_{0}+T_{s}}\left[w_{i}\left(t\right)+jw_{q}\left(t\right)\right]e^{-j2\pi (f+kf_{s})t}\;dt.$$

Equation (36) represents an exact, continuous-time relation for extracting the harmonic coefficients. The simplified waveform derivations presented below are used to interpret specific values of $H_{0}\left(f\right)$ under the large-RC approximation.

**Figure 11:**
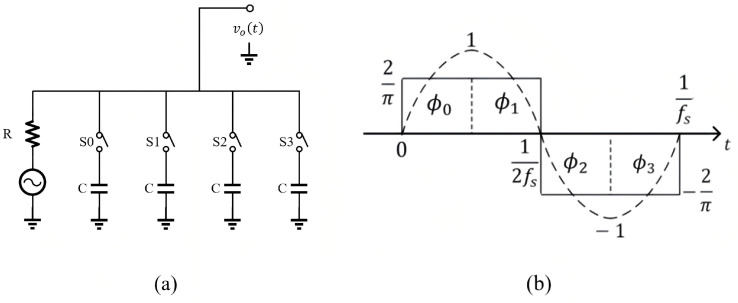
(**a**) Four-path filter. (**b**) The input (dashed line) and the four-phase outputs $\phi_{0-3}$ (solid line).

For the four-path example, assume $RC\gg T_{s}/4$, so the capacitor voltage changes only slightly during one switching interval and can be approximated by the average of the applied waveform. At dc, each capacitor settles to the input level, and $H_{0}\left(0\right)=1$. At $f_{in}=f_{s}$, consider the sine excitation in Figure 11. During the first on interval, the normalized capacitor voltage is approximated by

(37) $$\bar{v}=\frac{4}{T_{s}}\int_{0}^{T_{s}/4}\sin\left(2\pi f_{s}t\right)\;dt=\frac{2}{\pi }.$$

**Figure 12:**
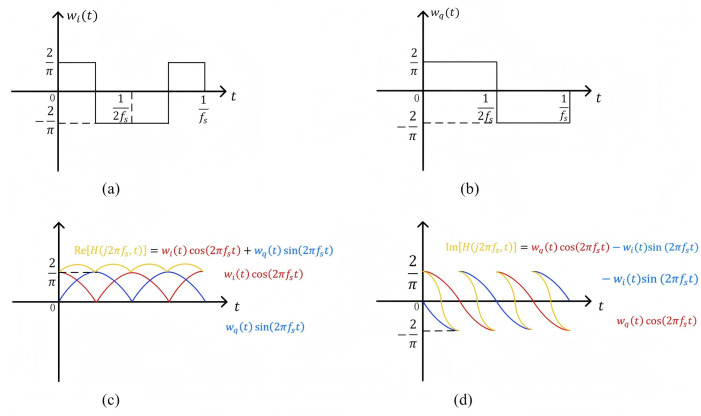
When $f_{in}=f_{s}$, (**a**) $w_{i}\left(t\right)$. (**b**) $w_{q}\left(t\right)$. (**c**) ReHf,t. (**d**) ImHf,t.

The four held capacitor values form the piecewise output shown in Figure 11b. Since the cosine and sine inputs differ by $T_{s}/4$, their outputs have the same relative shift. Equations (32) and (33) then produce the real and imaginary parts shown in Figure 12. The imaginary part has zero period average, while the real part gives

(38) Re[H(fs,t)]=2π2+2π2=8π2.

**Figure 13:**
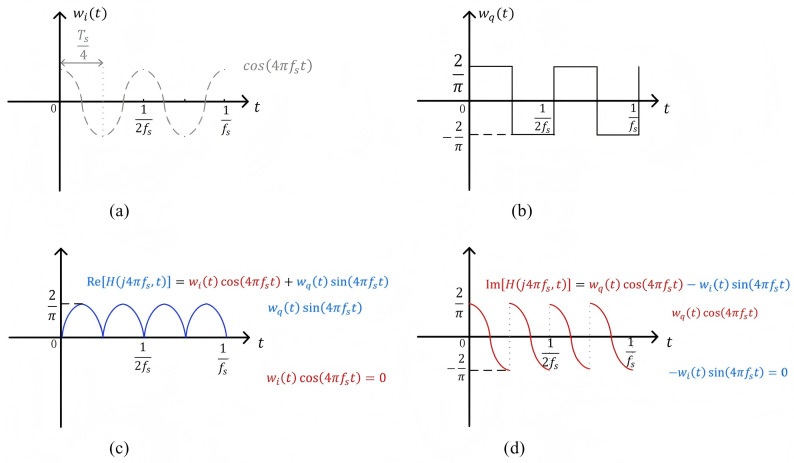
When $f_{in}=2f_{s}$, (**a**) $w_{i}\left(t\right)$. (**b**) $w_{q}\left(t\right)$. (**c**) ReHf,t. (**d**) ImHf,t.

Thus, $H_{0}\left(f_{s}\right)=8/\pi^{2}$. Repeating the same construction at $f_{in}=2f_{s}$ gives the waveforms in Figure 13 and

(39) Im[H(2fs,t)]=0Re[H(2fs,t)]=2π2=4π2

Hence, $H_{0}\left(2f_{s}\right)=4/\pi^{2}$. These limiting values are consistent with the established switched-RC analyses in [26,27,30].

**Figure 14:**
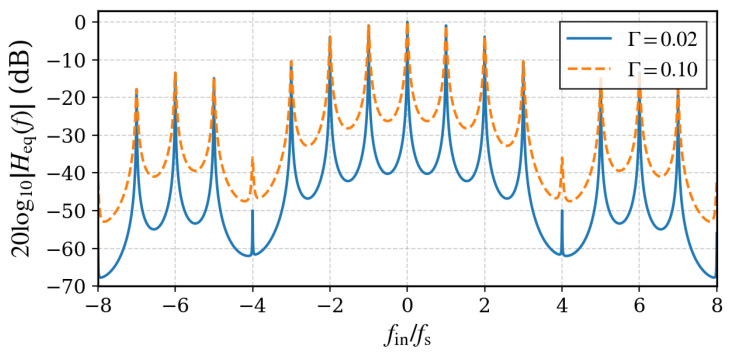
Equivalent sampled response magnitude $|H_{\mathrm{eq}}\left(f\right)|$ for Γ=τ/(RC)=0.02 and 0.10.

Figure 14 compares the linear magnitudes of the equivalent sampled responses for two values of Γ. Because Γ=τ/(RC), increasing Γ corresponds to reducing RC relative to the switch-on interval and therefore weakens the capacitor averaging behavior. At the nominal null frequencies $4f_{s}$ and $8f_{s}$, the residual magnitudes increase from 0.00318 and 0.00159 for Γ=0.02 to 0.01592 and 0.00796 for Γ=0.10, respectively. These values correspond to −49.94 dB, −55.96 dB, −35.96 dB, and −41.98 dB. Thus, a smaller Γ (larger RC) more closely approaches the ideal averaging limit and produces deeper nulls, whereas a larger Γ increases the finite RC error.

**Figure 15:**
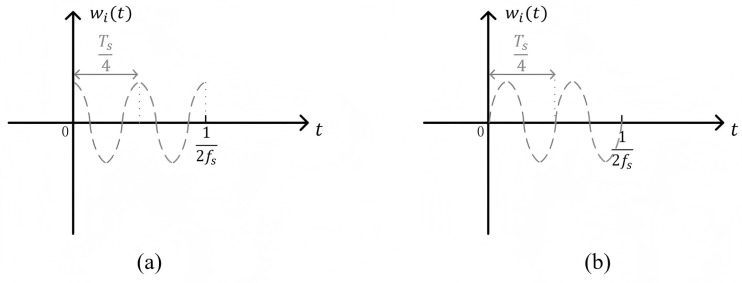
Average value equals zero for (**a**) $\cos\left(8\pi f_{s}t\right)$, (**b**) $\sin\left(8\pi f_{s}t\right)$.

At $f_{\mathrm{in}}=Nf_{s}=4f_{s}$, the input completes one full cycle during each interval of length $T_{s}/4$. Its average over every active interval is therefore zero, as illustrated in Figure 15, and the large-RC approximation gives $H_{0}\left(4f_{s}\right)=0$. More generally, an input at $f_{\mathrm{in}}=kNf_{s}$ completes *k* full cycles during each path interval, so $H_{0}\left(kNf_{s}\right)=0$ in the ideal averaging limit.

#### Quantitative Consistency Check

To quantify the accuracy and applicability of our time-domain interpretation, we numerically integrated Equation (1) for an ideal four-path network. We set the normalized parameters to $T_{s}=1$, $\tau =T_{s}/4$, and Γ=τ/(RC)=0.02. A complex sinusoidal input was represented by separate cosine and sine runs, and we extracted the harmonic coefficients using Equation (36). The simulation used 2000 time steps per switching period and ran for 500 periods to ensure steady state. Doubling the time resolution changed the reported magnitudes by less than 0.05%.

Table 3 compares the extracted numerical $H_{0}$ values with the intuitive large-RC limits in the decibel domain. At $f_{s}$ and $2f_{s}$, the magnitude differences remain below 0.001 dB. At the nominal null frequencies, the finite value of Γ produces residuals of −49.95 dB at $4f_{s}$ and −55.97 dB at $8f_{s}$, both normalized to the dc response. These results support our physical interpretation, while demonstrating that the exact null is an asymptotic limit of the averaging approximation.

Equation (36) also permits a direct verification of the higher-order coefficients. Table 4 lists the coefficients extracted at $f_{\mathrm{in}}=f_{s}$ in decibels. Only indices that are integer multiples of four remain, as predicted by the N-path principle in Equation (13). All nonmultiples of four within the range −8≤k≤8 remain below −273 dB in the normalized calculation. The time-domain interpretation therefore extends consistently to higher-order HTFs when using the full coefficient integral, although the simple averaging arguments remain most transparent for the fundamental component $H_{0}$. A comparison of the four analysis methodologies is summarized in Table 5.

## 4. Conclusions

This tutorial review places the principal N-path filter analysis methods within a unified LPTV framework. Continuous-time window function analysis provides the most complete harmonic description but requires substantial algebra. Discrete-time ODE analysis yields a compact sampled response, although it does not directly expose every translated component. Adjoint network analysis offers an efficient route to find the equivalent sampled impulse response. The orthogonal-excitation viewpoint serves a different purpose, providing a physically transparent interpretation of capacitor averaging, path-phase cancellation, $H_{0}\left(f\right)$, and the gain-null condition at $f_{\mathrm{in}}=kNf_{s}$. This article deliberately positions the orthogonal-excitation viewpoint as an educational interpretation rather than a new HTF theory. The numerical integration of the ideal switched-RC equations confirms the limiting values at $f_{s}$ and $2f_{s}$ to within 0.001 dB for Γ=0.02. It also quantifies the finite-RC residuals at the nominal null frequencies. The general coefficient–extraction relationship shows how the same orthogonal responses recover higher-order HTFs. Together, our review and consistency checks clarify the utility and boundaries of the time-domain picture. They guide readers in selecting the appropriate analytical method based on their target output quantity and accuracy requirements.

## Figures and Tables

**Table 1:** Derivation model, time-domain equation, and transfer function for continuous-time output in [23].

Derivation model	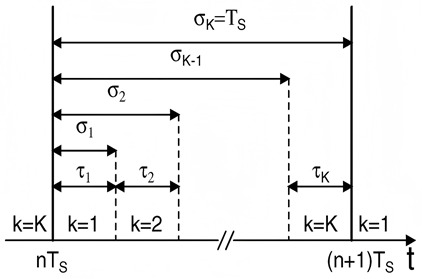
Time-domain equation	$v_{i,k}\left(t\right)=v_{o,k}\left(t\right)+RC\frac{dv_{o,k}\left(t\right)}{dt}$ $\sum_{n=-\infty }^{\infty }F\left(v_{0}\left(t\right)\right)\delta \left(t-nT_{s}-\sigma_{k}\right)$ $=\sum_{n=-\infty }^{\infty }\left[G_{k}\left(f\right)\cdot F\left(v_{i}\left(t\right)\right)\right]*\delta \left(f-nf_{s}\right)e^{-j2\pi nf_{s}\sigma_{k}}$
Transfer function	$V_{o}\left(f_{o}\right)=\sum_{n=-\infty }^{\infty }H_{n}\left(f_{o}\right)V_{i}\left(f_{o}-nf_{s}\right)$ $H_{n}\left(f_{o}\right)=\frac{f_{rc}}{jf_{o}+f_{rc}}\left[\frac{1-e^{-j2\pi nf_{s}\tau_{1}}}{j2\pi nf_{s}}+f_{s}G_{o}\left(f_{o}-nf_{s}\right)\frac{e^{j2\pi \frac{-f_{o}}{f_{s}}\tau_{2}}-1}{j2\pi f_{o}}\right]$ $G_{o}\left(f\right)=\frac{e^{j2\pi f\tau_{1}}-e^{-j2\pi f(T_{s}-\tau_{1})}}{e^{j2\pi fT_{s}}-1}\frac{1}{1+j\frac{f}{f_{rc}}}$

**Table 2:** Derivation model, time-domain equation, and transfer function for discrete-time output in [22].

Derivation model	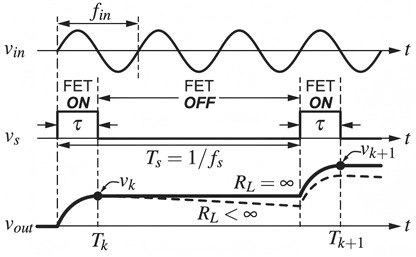
Time-domain equation	$v_{k+1}-v_{k}=\frac{1}{C}\int_{T_{k}}^{T_{k+1}}\frac{v_{in}\left(t\right)-v_{out}\left(t\right)}{R}dt.$
	$v_{out}\left(t\right)=\frac{1}{C}\int_{T_{k}+\tau }^{t}i\left(t^{\prime }\right)dt^{\prime }+v_{k}$
	$Ri\left(t\right)+v_{out}\left(t\right)=v_{in}\left(t\right)$
Transfer function	$H^{*}\left(j\omega_{in}\right)≜\frac{V_{out}^{*}\left(j\omega_{in}\right)}{V_{in}^{*}\left(j\omega_{in}\right)}=\frac{1}{1+j\omega_{in}RC}\frac{1-e^{-\tau /(RC)}e^{-j\omega_{in}\tau }}{1-e^{-\tau /(RC)}e^{-j\omega_{in}T_{s}}}$

**Table 3:** Decibel-domain comparison of the intuitive and numerical $H_{0}$ magnitudes for a four-path filter with Γ=0.02.

$f_{\mathrm{in}}/f_{s}$	Intuitive $20\log_{10}\left|H_{0}\right|$ (dB)	Numerical $20\log_{10}\left|H_{0}\right|$ (dB)	Absolute Difference or Residual (dB)
0	0.000	0.000	<0.001
1	−1.824	−1.825	<0.001
2	−7.845	−7.845	<0.001
4	−∞	−49.95	−49.95
8	−∞	−55.97	−55.97

**Table 4:** Selected higher-order HTF conversion gains in decibels for a fixed input at $f_{\mathrm{in}}=f_{s}$.

*k*	−8	−4	0	4	8
$f_{\mathrm{out}}/f_{s}=1+k$	−7	−3	1	5	9
$20\log_{10}\left|H_{k}\left(f_{s}\right)\right|$ (dB)	−18.73	−11.37	−1.82	−15.80	−20.91

*Note:* For the fixed input tone $f_{\mathrm{in}}=f_{s}$, the coefficient $H_{k}\left(f_{s}\right)$ is the harmonic conversion gain from $f_{\mathrm{in}}$ to $f_{\mathrm{out}}=f_{\mathrm{in}}+kf_{s}=\left(k+1\right)f_{s}$. Therefore, the entries at positive and negative *k* correspond to different output frequencies and are not required to be symmetric about k=0. For a real LPTV network, conjugate symmetry relates $H_{k}\left(f\right)$ to $H_{-k}^{*}\left(-f\right)$ rather than generally relating $H_{k}\left(f\right)$ to $H_{-k}\left(f\right)$.

**Table 5:** Comparison of N-path filter analysis methodologies.

Comparison Dimension	Continuous-Time Window Function [27]	Discrete-Time ODE [26]	Adjoint Network [30]	Time-Domain Interpretation
Mathematical basis	State-space equations combined with window-spectrum convolution	Switched-RC ordinary differential equations evaluated at the switching instants	Equivalent sampled linear time-invariant impulse response	Orthogonal excitation and Fourier-coefficient extraction
Main output	Complete continuous-time harmonic transfer functions	Compact sampled-domain or same-frequency response	Equivalent sampled transfer function	Physical interpretation of $H_{0}$ and direct extraction of general $H_{k}$
Main advantage	Captures frequency-translated components and higher-order harmonics	Avoids explicit convolution with the switching window spectrum	Simplifies the calculation of periodically sampled responses	Provides transparent links among capacitor averaging, phase cancellation, and HTFs
Main limitation	Relatively high algebraic and computational complexity	Does not directly provide all frequency-translated components	Primarily formulated for periodically sampled linear networks	Simple waveform arguments require $RC\gg T_{s}/4$; the full integral formulation is required for general $H_{k}$

## Data Availability

All data supporting this study were generated through numerical simulations and are provided in the article.

## References

[1] Molnar A., Andrews C. Impedance, filtering and noise in n-phase passive CMOS mixers. Proceedings of the IEEE 2012 Custom Integrated Circuits Conference.

[2] Darvishi M., van der Zee R., Nauta B. (2013). Design of Active N-Path Filters. IEEE J. Solid-State Circuits.

[3] Thomas C.M., Larson L.E. (2015). Broadband Synthetic Transmission-Line N-Path Filter Design. IEEE Trans. Microw. Theory Tech..

[4] Liu M. (2006). Demystifying Switched-Capacitor Circuits.

[5] Barber N.F. (1947). Narrow Band-Pass Filter Using Modulation. Wirel. Eng..

[6] Yin Y., Qi H., Lu H., Feng Z., He J., Zhang X., Li L., Qi X., Feng X. (2025). A Novel Active Polyphase Filter Employing Frequency-Dependent Image Rejection Enhancement Technique. Micromachines.

[7] Abidi A.A. (2007). The Path to the Software-Defined Radio Receiver. IEEE J. Solid-State Circuits.

[8] Andrews C., Molnar A.C. (2010). A Passive Mixer-First Receiver with Digitally Controlled and Widely Tunable RF Interface. IEEE J. Solid-State Circuits.

[9] Ghaffari A., Klumperink E.A.M., Soer M.C.M., Nauta B. (2011). Tunable High-Q N-Path Band-Pass Filters: Modeling and Verification. IEEE J. Solid-State Circuits.

[10] Lien Y.C., Klumperink E.A.M., Tenbroek B., Strange J., Nauta B. (2019). High-linearity bottom-plate mixing technique with switch sharing for N-path filters/mixers. IEEE J. Solid-State Circuits.

[11] Klumperink E.A., Westerveld H.J., Nauta B. N-path filters and mixer-first receivers: A review. Proceedings of the 2017 IEEE Custom Integrated Circuits Conference (CICC).

[12] Darabi H., Mirzaei A., Mikhemar M. (2011). Highly Integrated and Tunable RF Front Ends for Reconfigurable Multiband Transceivers: A Tutorial. IEEE Trans. Circuits Syst. I Regul. Pap..

[13] Soer M.C.M., Klumperink E.A.M., Ru Z., van Vliet F.E., Nauta B. A 0.2-to-2.0GHz 65nm CMOS receiver without LNA achieving ≫11dBm IIP3 and ≪6.5 dB NF. Proceedings of the 2009 IEEE International Solid-State Circuits Conference—Digest of Technical Papers.

[14] Andrews C., Molnar A.C. A passive-mixer-first receiver with baseband-controlled RF impedance matching, ≪6dB NF, and ≫27dBm wideband IIP3. Proceedings of the 2010 IEEE International Solid-State Circuits Conference—(ISSCC).

[15] Cook B.W., Berny A., Molnar A., Lanzisera S., Pister K.S.J. (2006). Low-Power 2.4-GHz Transceiver with Passive RX Front-End and 400-mV Supply. IEEE J. Solid-State Circuits.

[16] Westerveld H., Klumperink E., Nauta B. A cross-coupled switch-RC mixer-first technique achieving +41dBm out-of-band IIP3. Proceedings of the 2016 IEEE Radio Frequency Integrated Circuits Symposium (RFIC).

[17] Shao H., Qi G., Mak P.I., Martins R.P. (2022). A 1.7–3.6 GHz 20 MHz-Bandwidth Channel-Selection N-Path Passive-LNA Using a Switched-Capacitor-Transformer Network Achieving 23.5 dBm OB-IIP_3_ and 3.4–4.8 dB NF. IEEE J. Solid-State Circuits.

[18] Shao H., Martins R.P., Mak P.I. 23.4 A 167 μW 71.7dB-SFDR 2.4GHz BLE Receiver Using a Passive Quadrature-Front-End, a Double-Sided Double-Balanced Cascaded Mixer and a Dual-Transformer-Coupled Class-D VCO. Proceedings of the 2024 IEEE International Solid-State Circuits Conference (ISSCC).

[19] Lin Z., Mak P.I., Martins R.P. (2014). Analysis and Modeling of a Gain-Boosted N-Path Switched-Capacitor Bandpass Filter. IEEE Trans. Circuits Syst. I Regul. Pap..

[20] Park J.W., Razavi B. (2014). Channel Selection at RF Using Miller Bandpass Filters. IEEE J. Solid-State Circuits.

[21] Mirzaei A., Darabi H., Murphy D. (2011). A Low-Power Process-Scalable Super-Heterodyne Receiver with Integrated High-*Q* Filters. IEEE J. Solid-State Circuits.

[22] Andrews C., Molnar A.C. (2010). Implications of Passive Mixer Transparency for Impedance Matching and Noise Figure in Passive Mixer-First Receivers. IEEE Trans. Circuits Syst. I Regul. Pap..

[23] Mirzaei A., Darabi H. (2011). Analysis of Imperfections on Performance of 4-Phase Passive-Mixer-Based High-Q Bandpass Filters in SAW-Less Receivers. IEEE Trans. Circuits Syst. I Regul. Pap..

[24] Mirzaei A., Darabi H., Leete J.C., Chen X., Juan K., Yazdi A. (2009). Analysis and Optimization of Current-Driven Passive Mixers in Narrowband Direct-Conversion Receivers. IEEE J. Solid-State Circuits.

[25] Yuan F., Opal A. Distortion analysis of periodically switched nonlinear circuits. Proceedings of the 1999 IEEE International Symposium on Circuits and Systems (ISCAS).

[26] Iizuka T., Abidi A.A. (2016). FET-R-C Circuits: A Unified Treatment—Part I: Signal Transfer Characteristics of a Single-Path. IEEE Trans. Circuits Syst. I Regul. Pap..

[27] Soer M.C.M., Klumperink E.A.M., de Boer P.T., van Vliet F.E., Nauta B. (2010). Unified Frequency-Domain Analysis of Switched-Series-*RC* Passive Mixers and Samplers. IEEE Trans. Circuits Syst. I Regul. Pap..

[28] Iizuka T., Abidi A.A. (2016). FET-R-C Circuits: A Unified Treatment—Part II: Extension to Multi-Paths, Noise Figure, and Driving-Point Impedance. IEEE Trans. Circuits Syst. I Regul. Pap..

[29] Schreier R., Temes G.C. (2005). Understanding Delta-Sigma Data Converters.

[30] Pavan S., Klumperink E. (2017). Simplified Unified Analysis of Switched-RC Passive Mixers, Samplers, and *N* -Path Filters Using the Adjoint Network. IEEE Trans. Circuits Syst. I Regul. Pap..

